# Acquired Cutaneous Lymphangiectasia: Dermoscopic Evidence from White-Yellowish Lacunae

**DOI:** 10.5826/dpc.1103a62

**Published:** 2021-07-08

**Authors:** Nicolás Silvestre-Torner, Adrián Imbernón-Moya, Marta Martínez-García, Fernando Burgos-Lázaro

**Affiliations:** 1 Department of Dermatology. Hospital Universitario Severo Ochoa. Avenida de Orellana, Leganés, Madrid, Spain; 2 Department of Pathology. Hospital Universitario Severo Ochoa. Avenida de Orellana, Leganés, Madrid, Spain

**Keywords:** Lymphangiectasia, Dermoscopy, Lymphedema, Breast cancer

## Case Presentation

**Figure 1:**
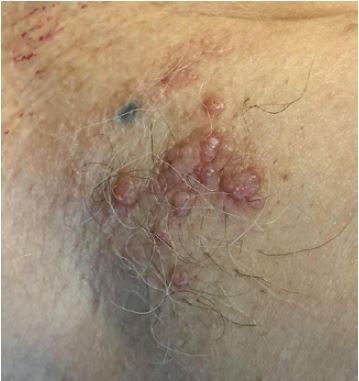
Multiple thin-walled papulovesicles on the left axillary area.

**Figure 2:**
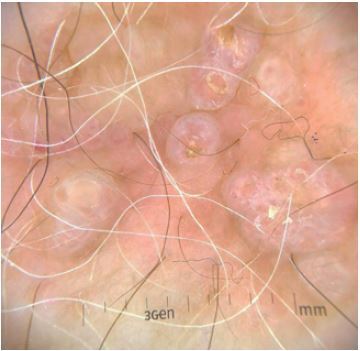
Dermoscopy revealing a vascular pattern with well-circumscribed yellowish lacunae surrounded by pale septa.

**Figure 3:**
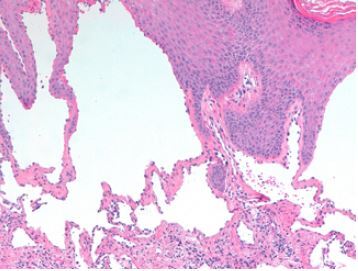
Histopathology revealing ectatic vessels in papillary dermis lined by a single layer of endothelial cells.

A 71-year-old woman, with a personal history of a left radical mastectomy and locoregional radiation therapy for breast cancer 20 years ago, was referred for assessment. She presented secondary chronic upper limb lymphedema and asymptomatic flesh-colored papulovesicles on the left axillary area (Figure 1) that appeared 6 months ago. On dermoscopy, lesions presented well-demarcated red-orange lacunae surrounded by white lines (Figure 2). Histopathology showed multiple ectatic lymphatic vessels in the papillary dermis (Figure 3). Thus, a diagnosis of acquired cutaneous lymphangiectasia was made.

## Teaching Point

Acquired cutaneous lymphangiectasia (ACL) are dilatations of surface lymphatic vessels, following lymphatic damage after surgery or radiotherapy, specially related with breast cancer [1]. Often described as “frog spawn”, ACL presents as multiple asymptomatic translucent vesicular lesions, resembling a lymphangioma circumscriptum. Dermoscopy shows a vascular pattern with yellow-orange lacunae surrounded by white septa [2]. Although ACL are considered benign disorders, histopathological diagnosis is needed to rule out different disorders, including cutaneous metastases from previous cancers.
